# The complete mitochondrial genome of Perciformes fish (*Brama dussumieri*) from South China Sea

**DOI:** 10.1080/23802359.2018.1501293

**Published:** 2018-08-13

**Authors:** Lei Xu, Xuehui Wang, Hong Li, Feiyan Du

**Affiliations:** a South China Sea Fisheries Research Institute, Chinese Academy of Fishery Sciences, Guangzhou, China;; b Guangdong Provincial Key Laboratory of Fishery Ecology and Environment

**Keywords:** Mitochondrial genome, *Brama dussumieri*, South China Sea

## Abstract

*Brama dussumieri* is one of the members of the family Bramidae and is widely distributed in the North Pacific Ocean. In this study, we described the complete mitochondrial genome of *B. dussumieri*. The genome is 16,996bp in length, encoding the standard set of 13 protein-coding genes, 22 tRNA genes, and 2 rRNA genes, with circular organization. The overall base composition of the whole mitochondrial genome was A (26.21%), T (25.65%), G (16.83%) and C (31.31%) with an AT bias of 51.86%. The ATG initiation codons are used in all protein-coding genes except *COX1*, and the stop codons of all the 13 protein-coding genes were complete.

The family Bramidae is a group of marine fishes belonging to the order Perciformes, which is widespread and occurrs in all tropical and temperate seas. Due to their characteristic body shapes, relatively large heads and high meristic counts of vertebrae and easily identifiable fin rays, the 22 recently identified species of the family Bramidae can be divided into seven genera within two subfamilies (Nelson 2006; Hatooka and Kai 2013; Lee et al. 2014). *Brama dussumieri* is widely distributed in the North Pacific Ocean (Lee and Kim 2015). It migrates seasonally between feeding and spawning grounds. Since late spring and through the summer periods, this species carries out a northward feeding migration along the subarctic frontal zone. Recently, the complete mitogenomes of *Brama japonica* in Bramidae have been reported (Chen et al. 2016). Here, we sequenced and annotate mitogenome of *B. dussumieri* form South China Sea to provide molecular information for genetically understanding of Perciformes fish.

The specimens of *B. dussumieri* were collected from the South China Sea (21°30′N, 117°30′E) on 15 September, 2017. Whole genomic DNA was extracted from muscle tissue of one specimen of *B. dussumieri* using TIANamp Marine Animals DNA Kit (TIANGEN, China). The concentration for use as a PCR template was adjusted to an A_260_ of about 0.05–0.2. The collected specimen and extracted DNA were stored in Guangdong Provincial Key Laboratory of Fishery Ecology and Environment. The complete mitochondrial genomes of *B. dussumieri* was sequenced using PCR primers designed from highly conserved regions of transfer RNA (tRNA) sequences of related species (Chen et al. 2016) with additional specific primers designed as required from sequences already obtained. Long-PCR amplifications were performed by thermo-cycling using five pairs of primers, and PCR amplicons were subjected to build up genomic library, and pair-end sequencing by MiSeq. The COI sequence of *B. dussumieri* was used as reference seeds for iterative assembly by MITObim v.1.8 (Hahn et al. 2013). SeqMan v.7.1.0 was used for the mitogenome assembly and annotation (Swindell and Plasterer 1997). Transfer RNA genes were predicted using online software tRNAScan-SE 1.21 (Lowe and Eddy 1997). All protein-coding genes (PCGs) are aligned independently, then concatenated to be applied for phylogenetic reconstruction with other Scombriformes in MrBayes v 3.12 (Ronquist and Huelsenbeck 2003) using relaxed clock model.

The *B. dussumieri* mitochondrial genome forms a 16,996 bp closed loop (GenBank accession number MH409959). The overall base composition of the whole mitochondrial genome was A (26.21%), T (25.65%), G (16.83%), and C (31.31%) with an AT bias of 51.86%. This mitochondrial genome represents a typical *Brama* mitochondrial genome and matches with the *B.japonica* genome, in which it comprises 13 protein-coding gene, 22 transfer RNA genes and 2 rRNA genes (12S rRNA and 16S rRNA) and 1 A + T-rich region which could also be termed as control region. The ATG initiation codon is used in all protein-coding genes except *COX1* (GTG), and the stop codons of all the 13 protein-coding genes were complete. Meanwhile, the longest protein-coding genes of these species was *ND5* (1839 bp), whereas the shortest was *ATP8* (168 bp). *lrRNA* and *srRNA* genes are of 1663bp and 956bp in length, respectively, and the length of D-loop is 1473 bp. All the 22 typical tRNAs possess a complete clover leaf secondary structure, ranging from 68 bp to 75 bp. The Bayesian inference phylogenetic tree showed that *B. dussumieri* firstly grouped with species of *B. japonica* (Figure 1). Phylogenetic tree indicates that *Brama* is not monophyly. We have the confidence to construct phylogenetic trees, based on the complete mitochondrial genomes, but the evolution history of flying fishes still needs future research to be clearly resolved.

**Figure 1.**
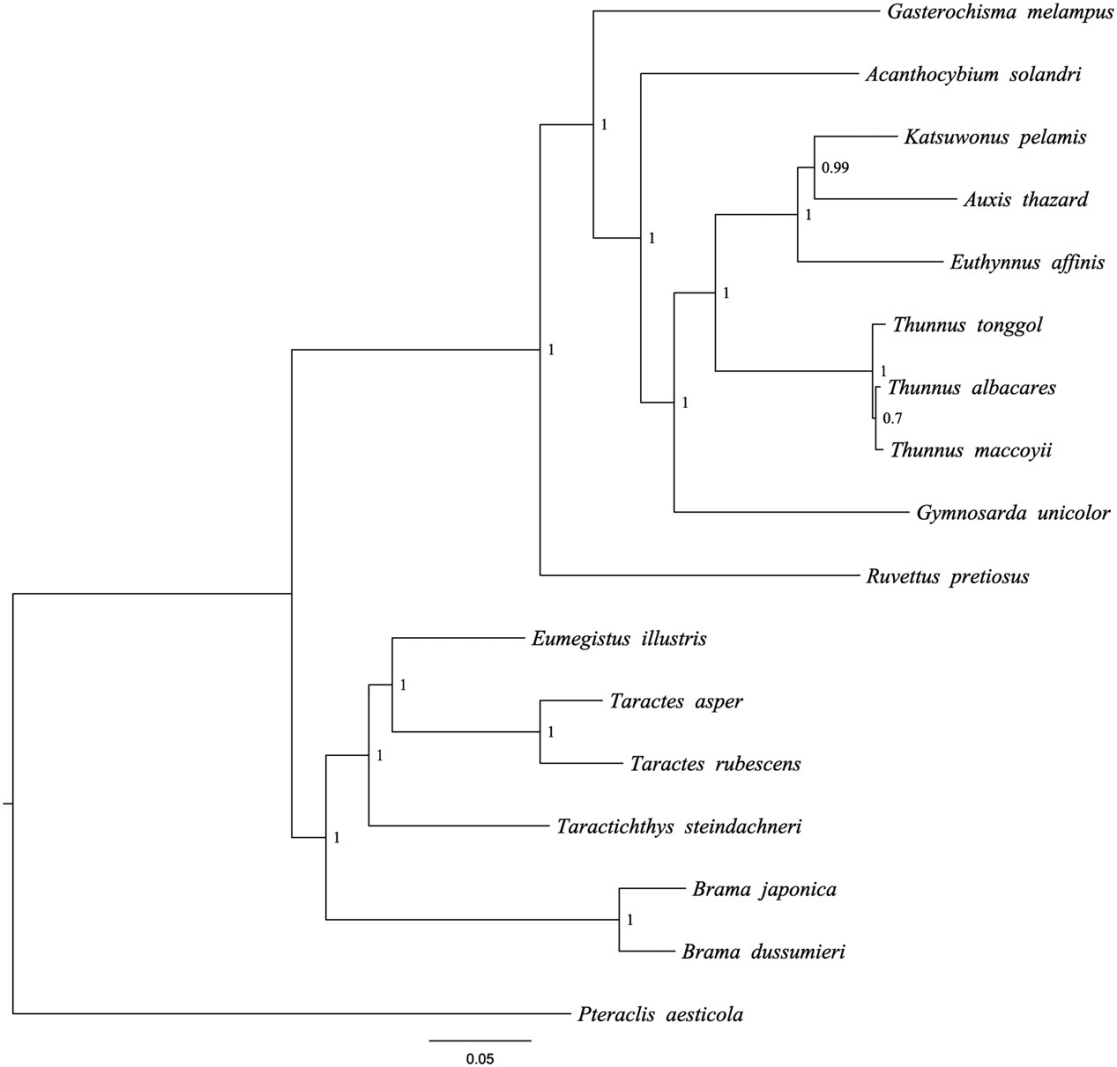
The Bayesian inference phylogenetic tree for Scombriformes based on mitochondrial PCGs and rRNAs concatenated dataset. The gene’s accession numbers for tree construction are listed as follows: *Brama japonica* (KT908039); *Eumegistus illustris* (AP012497); *Taractichthys steindachneri* (KT153629); *Taractes asper* (AP012498); *Taractes rubescens* (KR349364); *Gasterochisma melampus* (AP006033); *Acanthocybium solandri* (AP012945); *Katsuwonus pelamis* (AB101290); *Thunnus tonggol* (HQ425780); *Ruvettus pretiosus*(AP012506); *Gymnosarda unicolor* (AP012510); *Thunnus albacares*(GU256528); *Thunnus maccoyii* (GU256523); *Pteraclis aesticola* (AP012499); *Euthynnus affinis* (AP012946); *Auxis thazard* (AB105447).
